# Spred-2 Deficiency Exacerbates Lipopolysaccharide-Induced Acute Lung Inflammation in Mice

**DOI:** 10.1371/journal.pone.0108914

**Published:** 2014-10-02

**Authors:** Yang Xu, Toshihiro Ito, Soichiro Fushimi, Sakuma Takahashi, Junya Itakura, Ryojiro Kimura, Miwa Sato, Megumi Mino, Akihiko Yoshimura, Akihiro Matsukawa

**Affiliations:** 1 Department of Pathology and Experimental Medicine, Graduate School of Medicine, Dentistry and Pharmaceutical Sciences, Okayama University, Okayama, Japan; 2 Department of Microbiology and Immunology, Keio University School of Medicine, Tokyo, Japan; University of Tokyo, Japan

## Abstract

**Background:**

Acute respiratory distress syndrome (ARDS) is a severe and life-threatening acute lung injury (ALI) that is caused by noxious stimuli and pathogens. ALI is characterized by marked acute inflammation with elevated alveolar cytokine levels. Mitogen-activated protein kinase (MAPK) pathways are involved in cytokine production, but the mechanisms that regulate these pathways remain poorly characterized. Here, we focused on the role of Sprouty-related EVH1-domain-containing protein (Spred)-2, a negative regulator of the Ras-Raf-extracellular signal-regulated kinase (ERK)-MAPK pathway, in lipopolysaccharide (LPS)-induced acute lung inflammation.

**Methods:**

Wild-type (WT) mice and *Spred-2^−/−^* mice were exposed to intratracheal LPS (50 µg in 50 µL PBS) to induce pulmonary inflammation. After LPS-injection, the lungs were harvested to assess leukocyte infiltration, cytokine and chemokine production, ERK-MAPK activation and immunopathology. For *ex* *vivo* experiments, alveolar macrophages were harvested from untreated WT and *Spred-2^−/−^* mice and stimulated with LPS. In *in* *vitro* experiments, specific knock down of *Spred-2* by siRNA or overexpression of *Spred-2* by transfection with a plasmid encoding the *Spred-2* sense sequence was introduced into murine RAW264.7 macrophage cells or MLE-12 lung epithelial cells.

**Results:**

LPS-induced acute lung inflammation was significantly exacerbated in *Spred-2^−/−^* mice compared with WT mice, as indicated by the numbers of infiltrating leukocytes, levels of alveolar TNF-α, CXCL2 and CCL2 in a later phase, and lung pathology. U0126, a selective MEK/ERK inhibitor, reduced the augmented LPS-induced inflammation in *Spred-2^−/−^* mice. Specific knock down of *Spred-2* augmented LPS-induced cytokine and chemokine responses in RAW264.7 cells and MLE-12 cells, whereas *Spred-2* overexpression decreased this response in RAW264.7 cells.

**Conclusions:**

The ERK-MAPK pathway is involved in LPS-induced acute lung inflammation. Spred-2 controls the development of LPS-induced lung inflammation by negatively regulating the ERK-MAPK pathway. Thus, Spred-2 may represent a therapeutic target for the treatment of ALI.

## Introduction

Acute lung injury (ALI) and its most severe form, acute respiratory distress syndrome (ARDS), remain major causes of morbidity and mortality in critically ill patients [1]. ALI/ARDS are characterized by massive leukocyte infiltration into the lung, which causes acute respiratory failure associated with severe inflammation and diffuse alveolar damage [2]. ALI/ARDS can occur as a result of many different clinical insults, including sepsis. In sepsis, acute respiratory failure is the consequence of a complex interaction of epithelial cells, endothelial cells and leukocytes with soluble factors, such as the bacterial endotoxin lipopolysaccharide (LPS) and endogenous cytokines [3]–[5]. Evidence indicates that excessive production of inflammatory cytokines is critical for the initiation and progression of ALI/ARDS, and may determine the clinical outcome in patients with ALI/ARDS [1], [6]. Therefore, controlling cytokine responses represents a new potential therapeutic approach.

There is great interest in studying the cellular processes and multifaceted signaling pathways involved in cytokine biology. When LPS is recognized by Toll-like receptor 4 (TLR4), several intracellular signaling pathways are activated, including the IκB kinase (IKK)-NF-κB and MAPK pathways [4], [7]. These signaling pathways in turn activate a variety of transcription factors, inducing many genes that encode inflammatory mediators [7]. The MAPK family is composed of the c-Jun N-terminal kinase (JNK)-1/2, p38 and extracellular signal-regulated kinase (ERK)-1/2 signaling pathways [8]. Activated MAPKs phosphorylate and activate numerous transcription factors that drive the production of various inflammatory cytokines. Recent studies showed that MAPKs are involved in the inflammatory response during lung injury. Inhibition of p38-MAPK reduced LPS-induced lung inflammation [9]. SP600125, a JNK inhibitor, or PD98059, a MEK/ERK inhibitor, reduced total protein and lactate dehydrogenase (LDH) activity in bronchoalveolar lavage (BAL) fluids, and diminished neutrophil influx into lungs [10]. U0126, a MEK/ERK inhibitor, efficiently attenuated LPS-induced pulmonary inflammation [11]. In murine acute lung inflammation induced by either LPS or lipid A, an active moiety of LPS, robust ERK and some p38 phosphorylation, but not JNK phosphorylation was observed [12].

Members of the Sprouty-related EVH1-domain-containing protein (Spred) protein family can act to inhibit Ras-dependent ERK signaling [13]. As the ERK-MAPK pathway is involved in LPS-induced acute lung inflammation, dysregulation of ERK-MAPK signaling by Spred proteins could affect LPS-induced ALI. However, the physiological functions of Spred proteins in lung pathology remain largely unknown. Spred-1 and -3 are selectively expressed in the brain and cerebellum, whereas Spred-2 is ubiquitously expressed in various tissues, including the lung [14], [15]. Here, we chose to focus on Spred-2 and investigate its role in the functional regulation of LPS-induced acute lung inflammation. We demonstrate for the first time that Spred-2 controls the development of LPS-induced lung inflammation by negatively regulating the ERK-MAPK pathway.

## Materials and Methods

### Reagents

For western blotting, antibodies to p44/42 MAPK (ERK1/2) and phospho-p44/42 MAPK (ERK1/2) were purchased from Cell Signaling Technology (Danvers, MA, USA). The antibodies used for immunohistochemistry included those against CD68 (Abcam, Cambridge, UK), pan-cytokeratin (Santa Cruz Biotechnology, Santa Cruz, CA, USA) and phospho-ERK1/2 (R&D Systems, Minneapolis, MN, UA). U0126 was purchased from Calbiochem (Merck KGaA, Darmstadt, Germany). RAW264.7 cells, a murine macrophage cell line, were purchased from RIKEN (Tokyo, Japan). MLE-12 cells, a murine lung epithelial cell line, were obtained from the American Type Culture Collection (ATCC: Manassas, VA, USA).

### Mice

*Spred-2^−/−^* mice that were backcrossed onto the C57BL/6J background have been previously reported [16], [17] and C57BL/6J mice were used as wild-type (WT) mice. These mice were bled and maintained under a continuous 12 hour light: 12 hour dark cycle in specific pathogen-free conditions at the Department of Animal Resources, Okayama University (Okayama, Japan). Male mice (8–12 weeks old) were used in this study. The mice were fed a standard laboratory diet and water *ad libitum*. The Animal Care and Use Committee at Okayama University approved all animal experiments conducted in this study, and all methods were carried out in accordance with the approved guidelines.

### LPS-induced acute lung inflammation

Mice were anesthetized with intraperitoneal injection of sodium pentobarbital, followed by ketamine HCL and intratracheally injected with 50 µL LPS (011B4: 1 mg/mL, Sigma–Aldrich, St. Louis, MO, USA) dissolved in PBS. In some experiments, mice were intranasally treated with 20 µL U0126 (5 mM) or vehicle control (DMSO) prior to LPS administration. At 6, 24 and 72 hours after LPS administration, mice were euthanized and BAL was harvested and centrifuged at 400×*g* for 6 min. Supernatants contained BAL fluids that were used for cytokine and chemokine measurements. Cell pellets were suspended in saline and cells were counted using a hemocytometer. Differential cell analysis was made after Giemsa staining of cytospin preparations. The left lobe of the lung was fixed with 4% paraformaldehyde and embedded in paraffin; 4 µm paraffin sections were stained with hematoxylin and eosin (H&E). The right lobe was immediately frozen in liquid nitrogen and used for ELISA and immunoblotting.

### Alveolar macrophage culture

Murine alveolar macrophages were isolated from non-treated mice. In brief, lungs were lavaged twice with 0.8 mL ice-cold PBS. The BAL was harvested, centrifuged at 300×*g* for 6 min at 4°C, and the cell pellets were suspended in RPMI-1640 media supplemented with 10% fetal bovine serum (FBS) and antibiotics. Cell viability was routinely greater than 95%, as determined by trypan blue exclusion. Cells were seeded in each well of 96-well plate at 5×10^4^ cells and incubated for 2 hours at 37°C in a 5% CO_2_ incubator. After incubation, the media was removed and cells were washed with cell culture media to remove non-adherent cells. The adherent alveolar macrophages were stimulated with 100 ng/mL of LPS for 24 hours. Supernatants were harvested and used for ELISAs.

### Short-interfering (si) RNA and plasmid transfection

A total of 1×10^6^ RAW264.7 cells and 2×10^6^ MLE-12 cells were transfected with 2 µg *Spred-2*-specific or non-targeting control siRNAs (Thermo Scientific, Yokohama, Japan) using an Amaxa nucleofector kit V (Lonza Cologne AG, Cologne, Germany) according to the manufacturer’s instructions and plated in a 24-well plate. After 18 hours, cells were stimulated with 100 ng/ml of LPS for 6 hours. Subsequently, culture supernatants were harvested and used for cytokine and chemokine ELISAs. The siRNA efficacy was validated by real-time quantitative PCR (RT-qPCR) using *Spred-2* primers. The expression was routinely 30% or less of the levels detected in the control cells. For plasmid transfections, RAW264.7 cells were seeded into 2 cm dishes at a density of 2×10^5^ cells per well and incubated overnight in a 5% CO_2_ incubator at 37°C. For each transfection, 16 µL Lipofectamine 2000 (Life Technologies, Carlsbad, CA, USA) and 8 µg *Spred-2* expression plasmid (Oligene, Rockville, MD) were added to 400 µL Opti-MEM (Life Technologies) and incubated for 5 minutes at room temperature before the mixtures were added to the cells. RAW264.7 cells that overexpressed *Spred-2* were grown in DMEM medium with 10% FBS for 4 hours, after which the cells were stimulated with 100 ng/mL of LPS for 6 hours.

### Real-time quantitative PCR (RT-qPCR)

Total RNA was isolated from the cultured cells and whole lungs using a High Pure RNA Isolation Kit or High Pure RNA Tissue Kit (Roche Applied Science, Penzberg, Germany), respectively. First-strand cDNA was constructed from 1 µg total RNA using oligo (dT)_12–18_ primers, and the cDNAs were used as templates for PCR. RT-qPCR analysis was performed using StepOne with Taqman PCR master mix (Applied Biosystems, Foster City, CA, USA). Primers (*Spred-2*, Mm01223872_g1) were purchased from Applied Biosystems. Gene expression was normalized using *GAPDH* expression as an internal control, and relative fold change values were calculated based on unstimulated or WT control group that were assigned an arbitrary value of 1.

### ELISA

Murine cytokines were measured using a standard sandwich ELISA methods, as previously described [18], [19]. The capture antibodies, detection antibodies and recombinant cytokines were purchased from R&D Systems. The ELISAs used in this study did not cross-react with other known murine cytokines. For lung cytokine measurements, lungs were homogenized in PBS containing 0.1% TritonX-100 and complete protease inhibitor (Roche) and centrifuged; the cleared supernatants were harvested and used for ELISAs.

### Immunoblot analysis

Cells or lung samples were lysed in lysis buffer (Cell Signaling), briefly sonicated, incubated on ice for 30 minutes, and centrifuged at 12,000 *g* for 10 minutes. Supernatants were collected and stored at –80°C until use. For protein fractionation, 10 µg cell lysate was loaded per lane on gels for fractionation by sodium dodecyl sulfate (SDS)-polyacrylamide gel electrophoresis (Life Technologies), and transferred onto a nitrocellulose membrane. After overnight incubation with a primary antibody, the membrane was counter-stained with horseradish peroxidase-conjugated anti-rabbit or -mouse IgG secondary antibody (Santa Cruz Biotechnology, Santa Cruz, CA, USA) and visualized with an enhanced chemiluminescence system (Cell Signaling). Blots were photographed, digitized and analyzed using Image J, a public domain software developed by the NIH.

### Immunohistochemistry

Immunostaining was carried out using the Histofine Simple Stain MAX-PO (Nichirei Biosciences Inc, Tokyo, Japan), according to the manufacturer’s instructions. In brief, sections (4 µm slices) were treated with 0.3% H_2_O_2_ in methanol and then incubated with anti-phospho-p44/42 MAPK (ERK1/2) overnight at 4°C. Sections were rinsed and incubated with peroxidase-labeled polymer at room temperature for 30 minutes. As a chromogen, diaminobenzidine (DAKO, Carpinteria, CA, USA) was used. For double staining immunohistochemistry, the first staining sections were washed with denaturing solution (Biocare Medical, Concord, CA, USA), rinsed and incubated with anti-CD68 or anti-pan-cytokeratin overnight at 4°C. Sections were rinsed and stained using Histofine SAB-AP (R) kit (Nichirei). First red (Nichirei) was used as a chromogen. Counter-staining was performed with hematoxylin.

### Statistics

Data were analyzed and plotted on graphs using GraphPad Prism 5.0b (GraphPad Software, San Diego, CA, USA). The statistical significance of differences between data sets was evaluated using ANOVA. Each data set was expressed as the mean ± SEM. Instances in which *P*<0.05 were considered to represent statistically significant differences.

## Results

### Spred-2 deficiency augments LPS-induced acute lung inflammation

**Figure 1:**
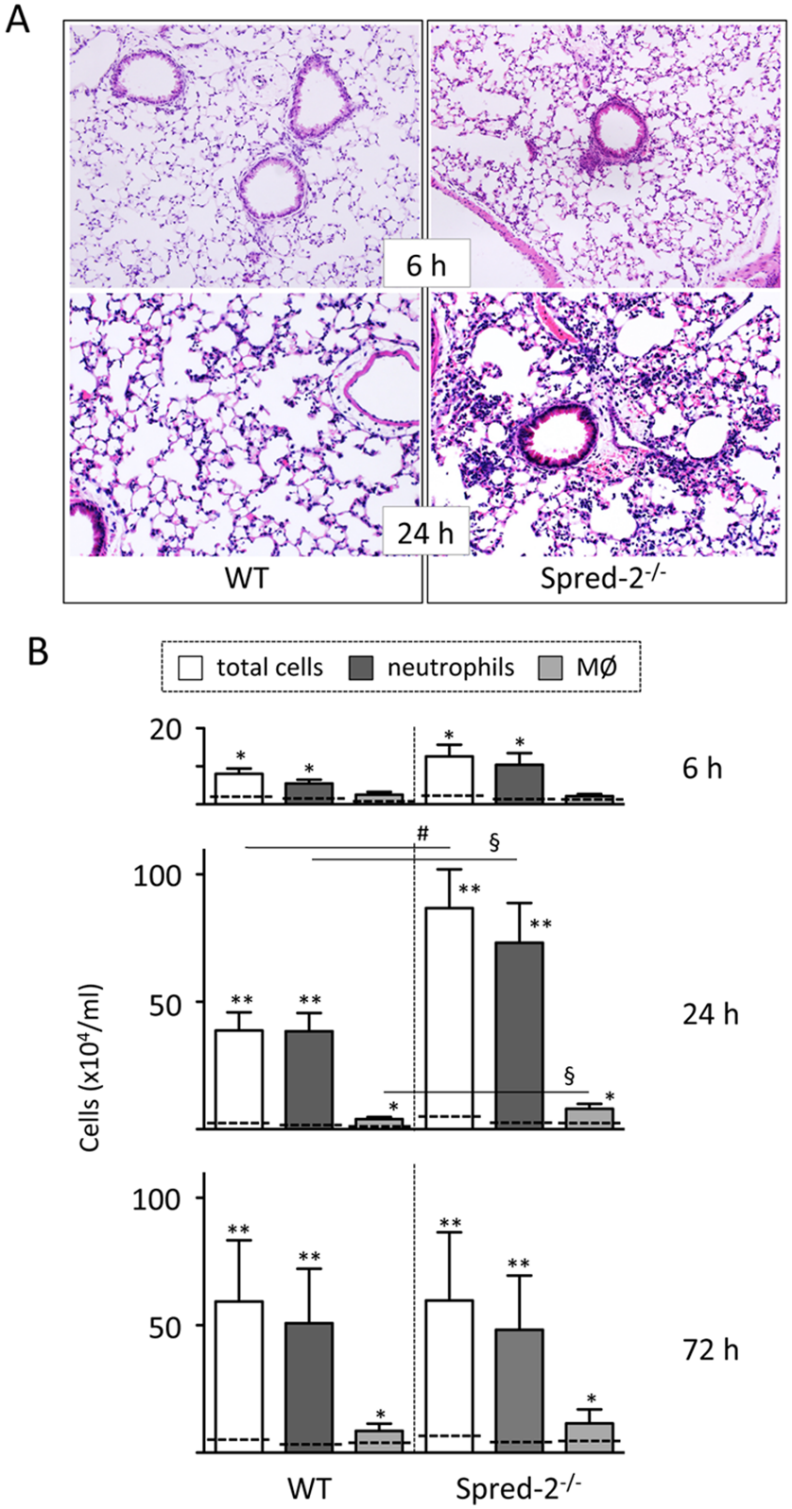
LPS-induced lung inflammation. Mice were injected intratracheally with LPS. (A) Representative images of lung sections (from groups of n = 4) after LPS administration are shown (original magnification, 200x). (B) The numbers of infiltrating leukocytes at 6, 24 and 72 hours time points in the lungs were counted (n = 6–7). *P<0.05, **P<0.01 vs. untreated mice. §*P*<0.05, #*P*<0.01, vs. WT control. Dotted lines were PBS treated controls (n = 3).

To understand the role of Spred-2 in LPS-induced acute lung inflammation, we employed *Spred-2^−/−^* mice and the mice were intratracheally injected with LPS. We confirmed that no *Spred-2* expression was detected in lungs from *Spred-2^−/−^* mice, as assessed by TaqMan RT-qPCR (data not shown). Analysis of lung H&E-stained sections showed that peribronchial neutrophil infiltration often occurred WT mice 6 hours after LPS administration, but was exacerbated in *Spred-2^−/−^* mice. At 24 hours, acute lung inflammation, characterized by peribronchiolar edema, infiltration of leukocytes with prominent neutrophils, and hemorrhage, was much more severe in *Spred-2^−/−^* mice than in WT mice (Fig. 1A). Significantly more leukocytes infiltrated the lungs of *Spred-2^−/−^* mice compared with WT mice at the peak (24 hours), including a 2.5-fold and 2.1-fold increase in the number of neutrophils and macrophages, respectively (Fig. 1B). There were no statistical differences at 6 and 72 hours after the injection. Thus, *Spred-2^−/−^* mice develop exacerbated acute pulmonary inflammation in a LPS-induced acute lung inflammation model.

### Altered production of cytokines and chemokines in *Spred-2^−/−^* mice

**Figure 2:**
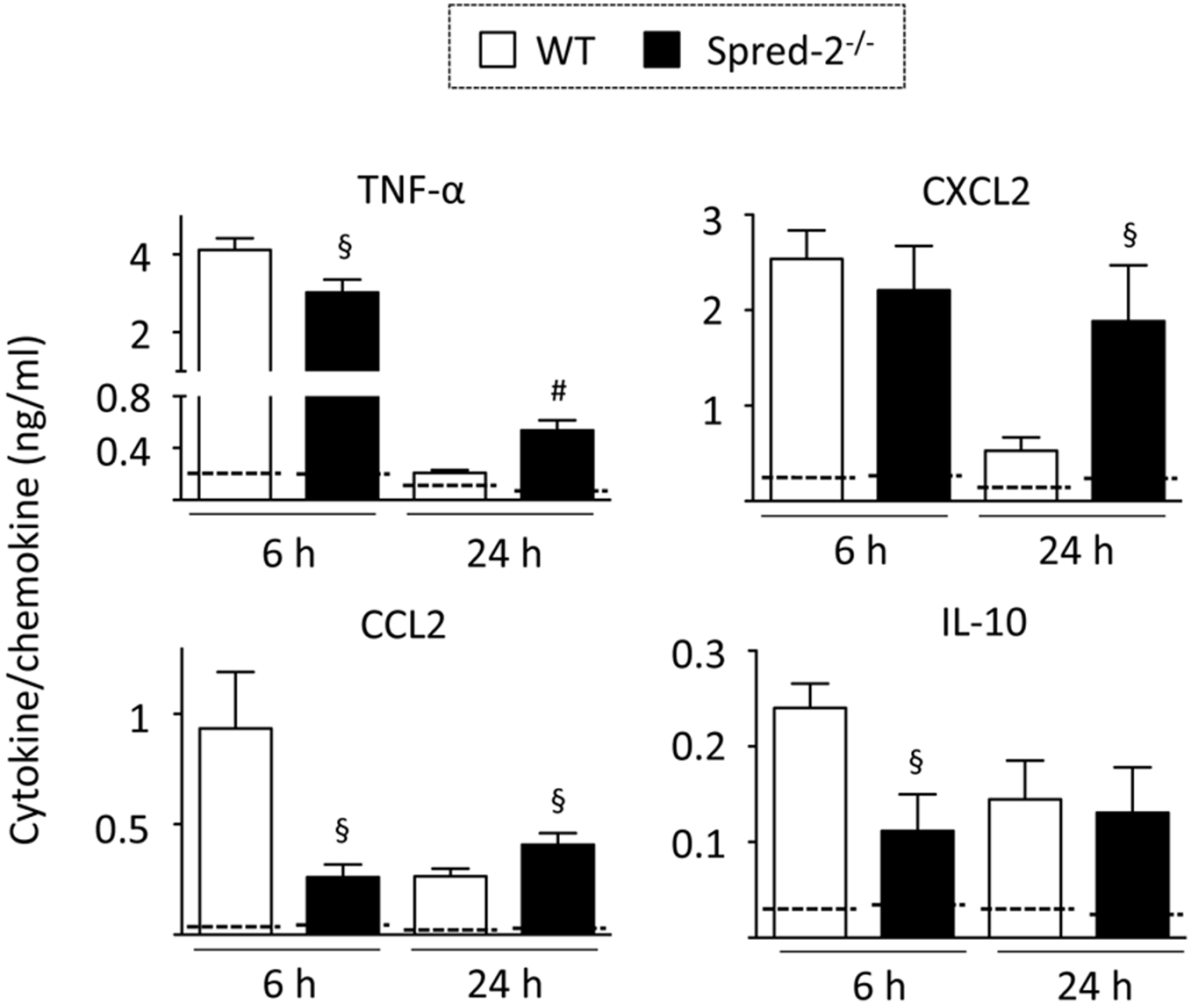
Cytokine and chemokine levels in LPS-induced acute lung inflammation. Mice were injected intratracheally with LPS (50 µg in 50 µL) or vehicle (PBS). At 6 and 24 hours after injection, mice were killed and BAL fluids were harvested from lungs of WT (6 hours, n = 6, 24 hours, n = 7) and *Spred-2^−/−^* mice (6 hours, n = 6, 24 hours, n = 9). TNF-α, CXCL2, CCL2 and IL-10 levels in the BAL fluids were measured by ELISA. §*P*<0.05, #*P*<0.01, vs. WT control. Dotted lines were PBS treated controls.

LPS induces the rapid production of proinflammatory cytokines and chemokines that can induce ALI in humans [20]. We next examined the cytokine and chemokine response in BAL fluids 6 and 24 hours after LPS administration. LPS provoked the production of the cytokine TNF-α and chemokines, such as CXCL2/MIP-2 and CCL2/MCP-1, in WT mice. As shown in Figure 2, levels of TNF-α and CCL2, but not CXCL2, at 6 hours were lower in *Spred-2^−/−^* mice than those in WT mice. Anti-inflammatory IL-10 level was also decreased in *Spred-2^−/−^* mice relative to WT mice. In contrast, levels of TNF-α, CXCL2 and CCL2 24 hours after LPS administration were significantly higher in *Spred-2^−/−^* mice than in WT mice. These data suggest that Spred-2 regulates the cytokine and chemokine response in this model.

### Enhanced ERK activation in *Spred-2^−/−^* mice

**Figure 3:**
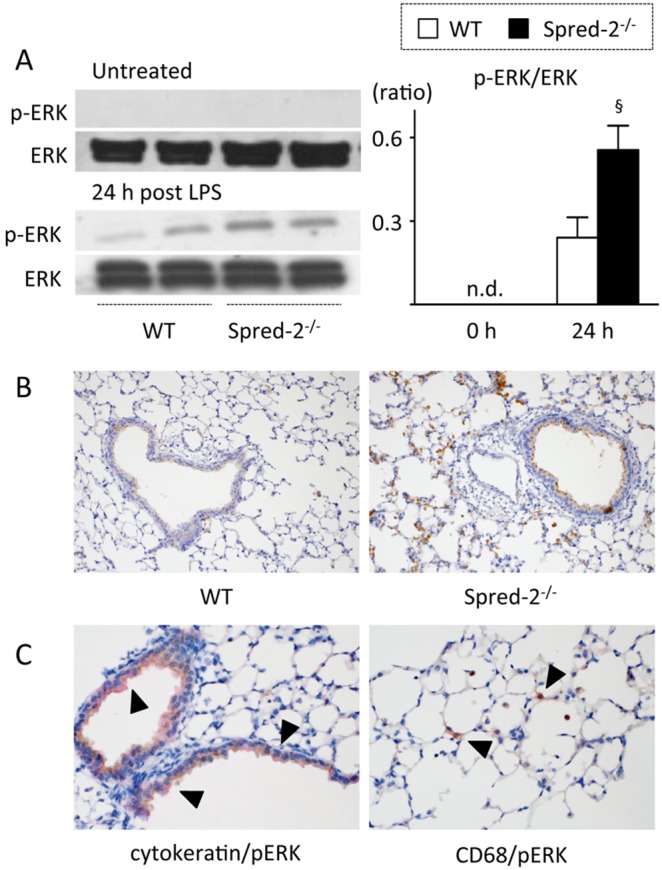
ERK activation in the lung after LPS administration. WT (n = 4) and *Spred-2^−/−^* mice (n = 3) were injected intratracheally with LPS (50 µg in 50 µL). At 24 hours after injection, mice were killed and lungs were harvested. Untreated WT and *Spred-2^−/−^* mice (n = 4) were used as controls. (A) Lung extracts were immunoblotted using the indicated primary antibodies. Left, representative immunoblot data from two independent analyses of lysates from different mice. Right, band densities were digitized and semi-quantitated. §*P*<0.05, vs. WT control. (B) Lung sections were stained with anti-pERK antibody (original magnification 200×). Representative images are shown. (C) Left, Cytokeratin and pERK positive cells were stained in red and brown, respectively. Right, CD68 and pERK positive cells were stained in red and brown, respectively. Arrows indicate double positive cells. Representative images are shown.

The key downstream pathway for LPS-induced signaling via TLR4 requires MAPKs [21]. The augmented acute lung inflammation in Spred-2 deficiency at the peak (24 hours) may be caused by enhanced cytokine and chemokine response at the time point through ERK-MAPK. To investigate the molecular basis for the augmented inflammatory response in *Spred-2^−/−^* mice, ERK activation in the lung was examined 24 hours after LPS administration. ERK activation, indicated by phosphorylated ERK molecules, was not detected in lungs from untreated WT and *Spred-2^−/−^* mice. After LPS administration, ERK phosphorylation occurred in all mice, but was significantly augmented in lungs from *Spred-2^−/−^* mice (Fig. 3A). By immunohistochemistry, the staining intensity of phosphorylated ERK was greater in lungs from *Spred-2^−/−^* mice than in WT controls (Fig. 3B). Notably, phosphorylated ERK could be detected in the cytoplasm of bronchial epithelial cells (cytokeratin positive cells) and alveolar macrophages (CD68 positive cells) (Fig. 3C). No staining was observed in the pneumocytes and peribronchiolar neutrophils (Fig. 3B). Thus, ERK activation was enhanced in *Spred-2^−/−^* mice after LPS administration.

### U0126 inhibits enhanced lung inflammation in Spred-2 KO mice

**Figure 4:**
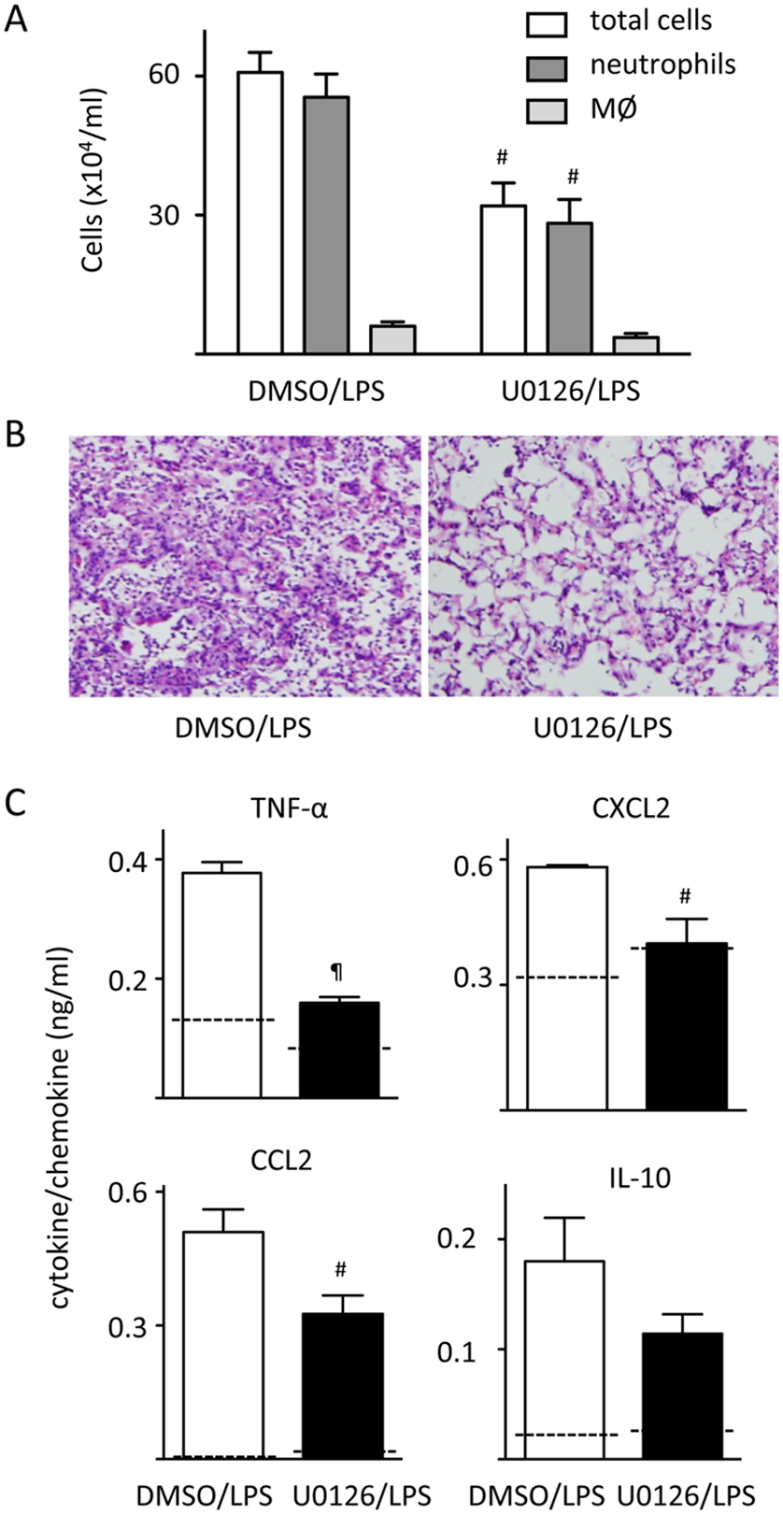
U0126 inhibits the enhanced lung inflammation in Spred-2^−/−^ mice. We treated *Spred-2^−/−^* mice intranasally with 20 µL of 5 mM U0126 (n = 6) or DMSO vehicle control (n = 5) before LPS administration (50 µg in 50 µL). At 24 hours after LPS administration, mice were killed. (A) The number of infiltrating leukocytes in BAL fluids was counted. #*P*<0.01, vs. WT mice. (B) Representative images of lung sections are shown (original magnification 200×). (C) TNF-α, CXCL2, CCL2 and IL-10 levels in BAL fluids were measured by ELISA. Dotted lines indicate the levels in BAL fluids from PBS-treated control mice. #*P*<0.01, ¶*P*<0.001, vs. WT control.

Our data suggested that mice with Spred-2 deficiency develop exacerbated lung inflammation as a result of enhanced Ras-ERK-MAPK signaling. To test this hypothesis, we administered U0126, an inhibitor of the ERK-MAPK pathway that blocks the kinase activity of MAP Kinase Kinase (MAPKK or MEK 1/2), 1 hour prior to LPS administration. We then harvested BAL fluids and lung tissues 24 hours after LPS administration. U0126 treatment reduced the enhanced neutrophil infiltration in *Spred-2^−/−^* mice (Fig. 4A). There was a trend towards a reduction in the number of macrophages (control vs. U0127, 6.05±0.95 vs. 3.58±0.88×10^4^ cells/mL, *P* = 0.09, n = 5). Likewise, U0126 dramatically ameliorated the exacerbated LPS-induced lung pathology observed in *Spred-2^−/−^* mice (Fig. 4B). The cytokine and chemokine response at 24 hours was next investigated in *Spred-2^−/−^* mice, and we found that U0126 significantly reduced the increased TNF-α, CXCL2 and CCL2 levels, but not IL-10, in *Spred-2^−/−^* mice (Fig. 4C). In WT mice, levels of TNF-α, CXCL2, CCL2 and IL-10 were not statistically different between DMSO/LPS and U0126/LPS groups (not shown). These results suggest that the enhanced leukocyte infiltration and cytokine responses observed in *Spred-2^−/−^* mice are largely dependent on enhanced Ras-ERK-MAPK activity.

### Augmented TNF-α, CXCL2 and CCL2 levels in *Spred-2^−/−^* alveolar macrophages

**Figure 5:**
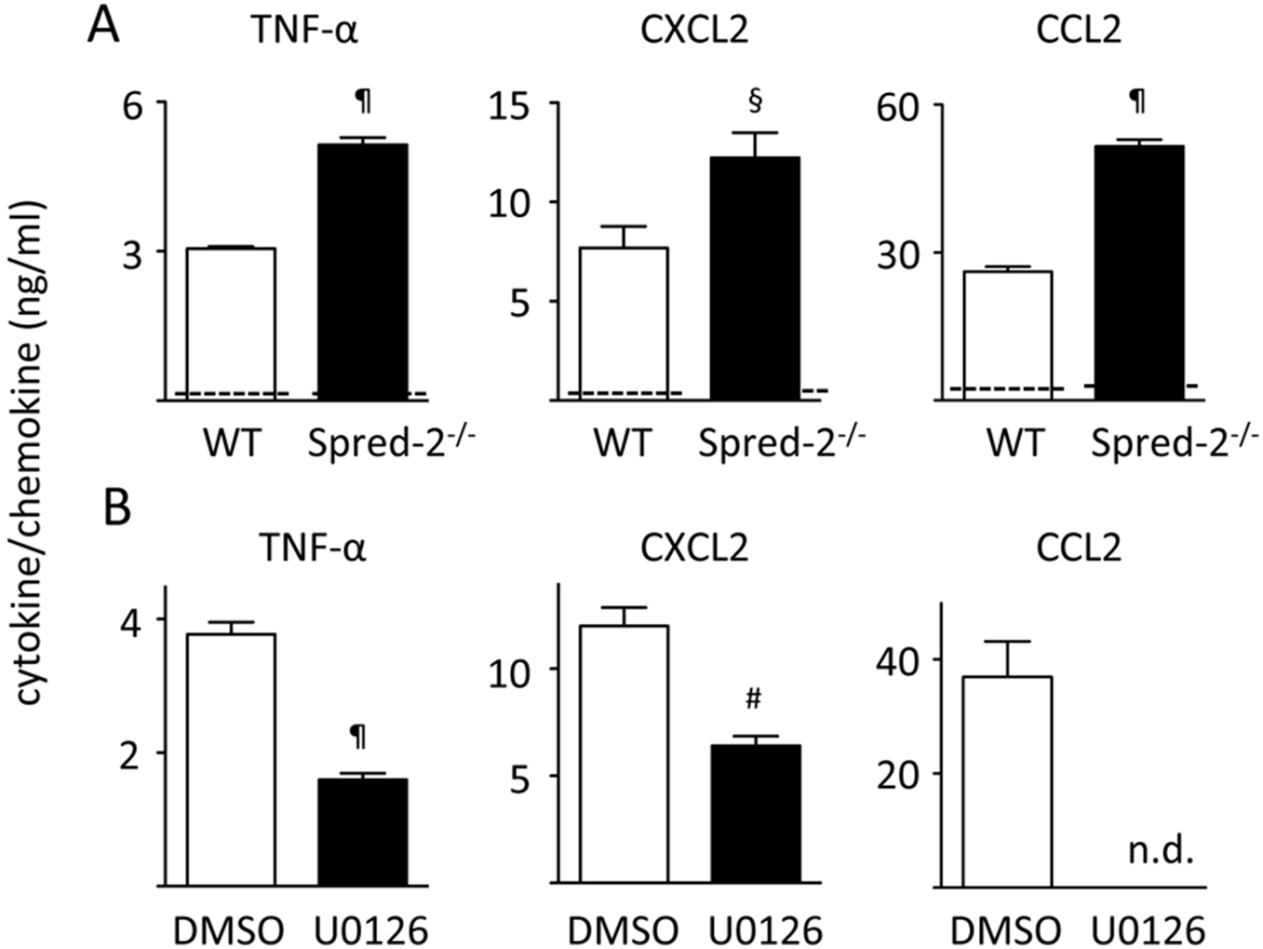
The production of TNF-α, CXCL2 and CCL2 by alveolar macrophages. (A) Alveolar macrophages were isolated from untreated WT and *Spred-2^−/−^* mice (n = 4–6) and stimulated with LPS (100 ng/mL) for 24 hours. Dotted lines indicate levels in alveolar macrophages from PBS treated control mice (n = 3). (B) Alveolar macrophages were isolated from untreated *Spred-2^−/−^* mice (n = 6) and stimulated with LPS (100 ng/mL) in the presence (n = 3) or absence (n = 3) of U0126 (10 µM) for 24 hours. TNF-α, CXCL2 and CCL2 levels were measured by ELISA. §*P*<0.05, #*P*<0.01, ¶*P*<0.001, vs. control DMSO. n.d., not detected.

**Figure 6:**
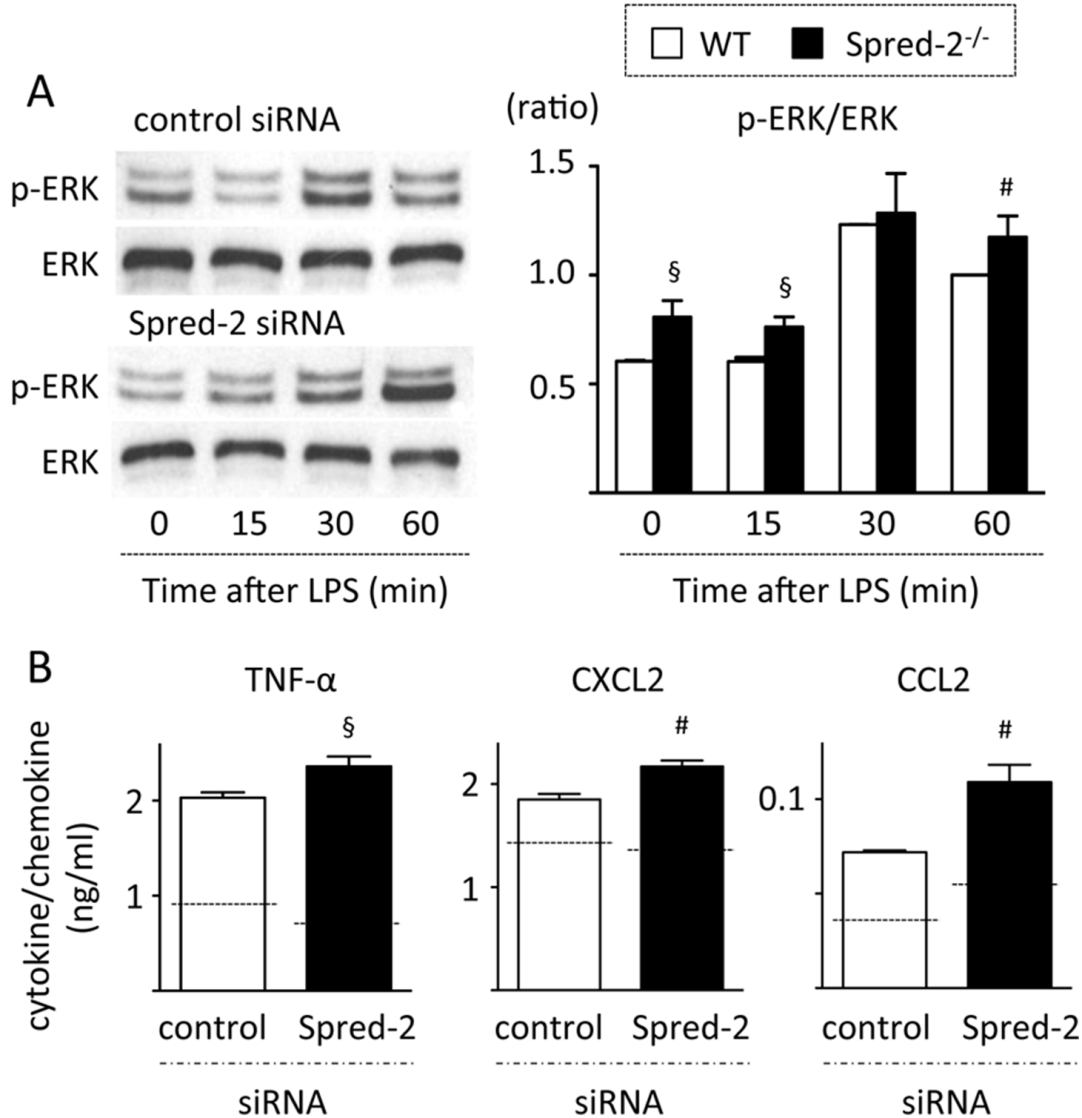
Knock down of Spred-2 in RAW264.7 cells. RAW264.7 cells were transfected with 2 µg *Spred*-2-specific or non-targeting control siRNA. After transfection, cells were stimulated with LPS (100 ng/mL) for the indicated time interval (A) or 6 hours (B). (A) Cells were extracted and immunoblotted with the indicated primary antibodies. Left, representative immunoblot data from 3 independent experiments. Right, band densities were digitized and semi-quantitated (n = 3). §*P*<0.05, #*P*<0.01, vs. control siRNA. (B) Cytokine levels in the culture supernatants were measured by ELISA. §*P*<0.05, #*P*<0.01, vs. control siRNA. Dotted lines indicate levels in PBS-treated control cells.

Alveolar macrophages are one of the major cell types that recognize pathogens and trigger inflammation by producing cytokines and chemokines [22]. We hypothesized that alveolar macrophages were responsible for the augmented inflammatory responses in *Spred-2^−/−^* mice. To test this hypothesis, alveolar macrophages were isolated from untreated WT and *Spred-2^−/−^* mice and the cells were stimulated with LPS for 24 hours. LPS-stimulated *Spred-2^−/−^* alveolar macrophages produced significantly higher levels of TNF-α, CXCL2 and CCL2 than WT alveolar macrophages (Fig. 5A). Similarly, bone marrow-derived macrophages from *Spred-2^−/−^* mice produced higher levels of TNF-α and CCL2 compared with the WT controls (Fig. S1). The levels of CXCL2 were not significantly different between the groups. The enhanced production of TNF-α, CXCL2 and CCL2 in alveolar macrophages from *Spred-2^−/−^* mice was inhibited by treatment with U0126 (Fig. 5B). When Spred-2 was “knocked down” by siRNA in RAW264.7 cells, ERK activation was slightly but significantly increased (Fig. 6A) and levels of TNF-α, CXCL2 and CCL2 were augmented compared with the control cells (Fig. 6B). These results suggested that alveolar macrophages contributed to the increased TNF-α, CXCL2 and CCL2 levels in *Spred-2^−/−^* mice.

### Epithelial cells contribute to the increased CCL2 levels

**Figure 7:**
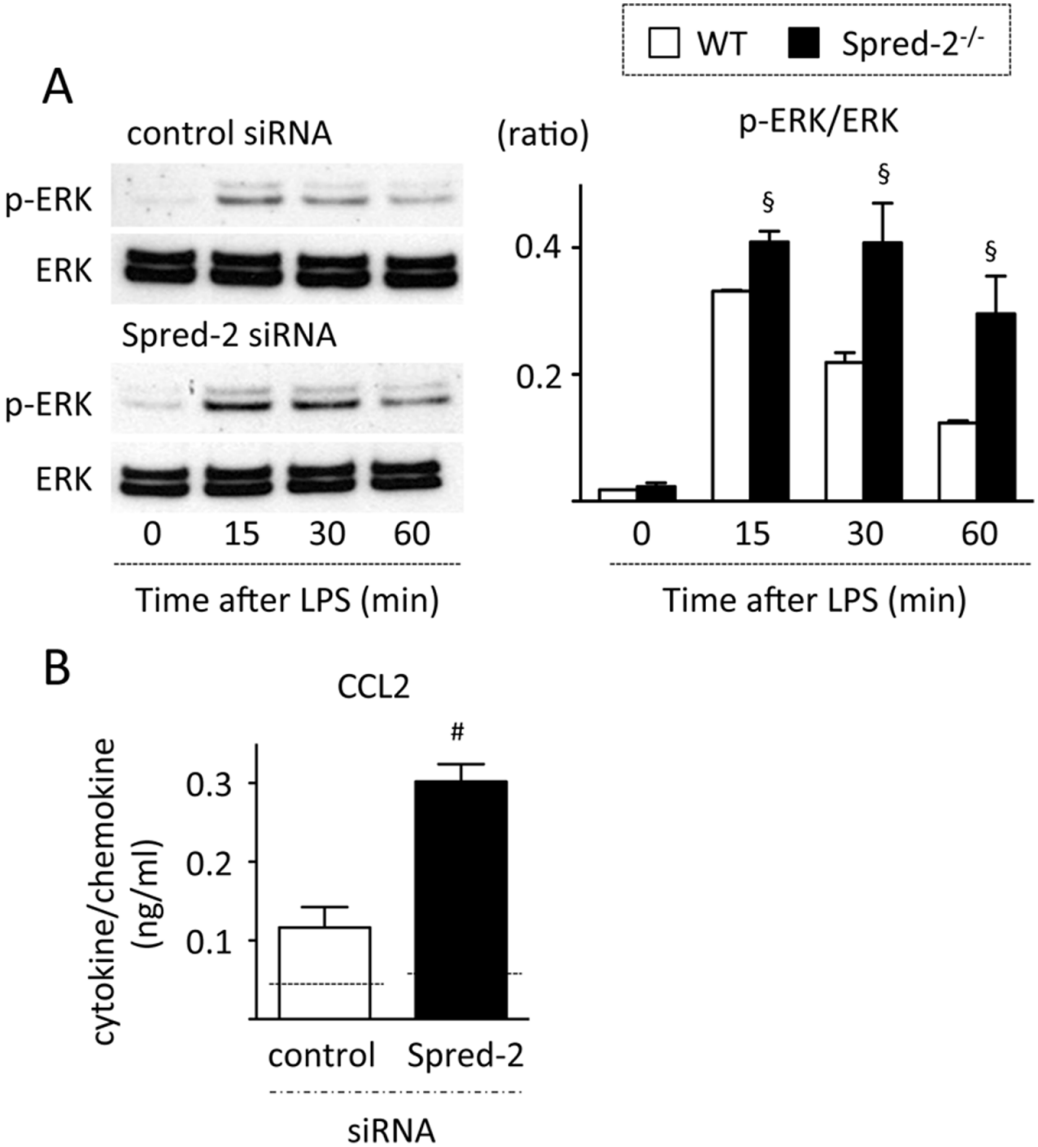
Knock down of Spred-2 in MLE-12 cells. MLE-12 cells were transfected with 2 µg *Spred-2*-specific or non-targeting control siRNA. After transfection, cells were stimulated with LPS (100 ng/mL) for the indicated time intervals (A) or 6 hours (B). (A) Cell lysates were extracted and immunoblotted with the indicated primary antibodies. Left, representative immunoblot data from 3 independent experiments. Right, band densities were digitized and semi-quantitated (n = 3). §*P*<0.05, #*P*<0.01, vs. control siRNA. (B) CCL2 levels in the culture supernatants were measured by ELISA. #*P*<0.01, vs. control siRNA. Dotted lines indicate levels in PBS-treated control cells.

Epithelial cells are another important cell type that secretes cytokines [5] and serve as physical and functional barriers to pathogens. To investigate whether epithelial cells contribute to the enhanced cytokine production in *Spred-2^−/−^* mice, MLE-12 cells in which *Spred-2* was specifically knocked down by siRNA and control cells were stimulated with LPS (Fig. 7A). *Spred-2* knock down resulted in increased levels of CCL2 compared with the control MLE-12 cells (Fig. 7B). Consistent with a published report [23], MLE-12 cells did not produce TNF-α after LPS stimulation. MLE-12 cells also failed to produce appreciable levels of CXCL2 (data not shown). Thus, epithelial cells are predicted to contribute to the augmented CCL2 levels, but not TNF-α and CXCL2 levels, in *Spred-2^−/−^* mice.

### *Spred-2* overexpression reduced LPS-induced cytokine responses

**Figure 8:**
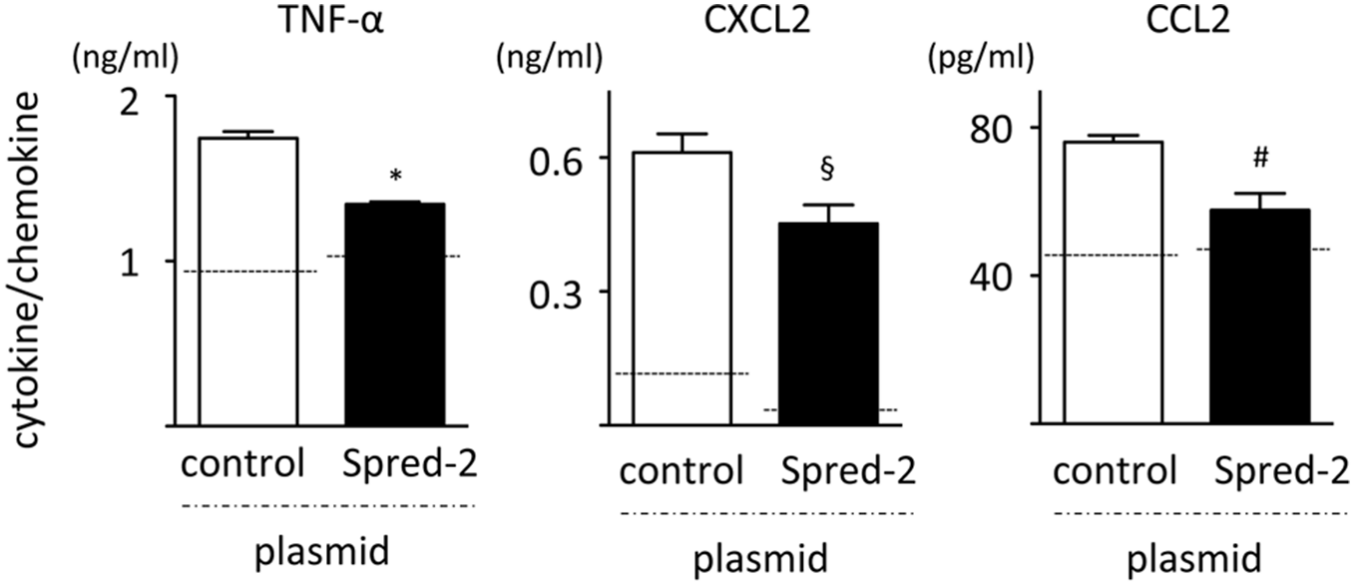
Overexpression of Spred-2 in RAW264.7 cells. *Spred-2* was overexpressed by transfecting RAW264.7 cells with mixtures of Lipofectamine 2000 and *Spred-2* overexpression plasmid for 24 hours, after which the cells were stimulated with LPS (100 ng/mL) for 6 hours (n = 3). Cytokine levels in the culture supernatants were measured by ELISA. §*P*<0.05, #*P*<0.01, *P<0.0001, vs. control siRNA. Dotted lines indicate levels in PBS-treated control cells.

The *Spred-2* knock down data raised the question of whether greater amounts of Spred-2 in a cell could reduce LPS-induced cytokine responses. To address this point, we overexpressed *Spred-2* in RAW264.7 cells using an overexpression plasmid that increased *Spred-2* expression by 20–25-fold, as determined by Taqman RT-qPCR (data not shown). *Spred-2* overexpression significantly reduced TNF-α, CXCL2 and CCL2 levels after LPS stimulation compared with the control cells (Fig. 8). Thus, Spred-2 overexpression effectively reduced LPS-induced cytokine responses. We did not carry out similar experiments using MLE cells because of the cell toxicity caused by the transfection of the plasmids.

## Discussion

ALI and ARDS are severe forms of diffuse lung disease that are associated with a high morbidity and mortality [24]. The LPS-induced lung inflammation model displays key features of lung injury seen in patients with ARDS [25] and is a widely used experimental model to investigate the mechanisms of acute lung injury. Here, we focused on Spred-2, a negative regulator of the ERK-MAPK pathway, in a murine model of LPS-induced acute lung inflammation and demonstrated for the first time that Spred-2 plays a protective role in this model. Mice with Spred-2 deficiencydemonstrated increased leukocyte infiltration and higher cytokine and chemokine levels at the peak time point (24 hours). As expected, ERK-MAPK activation was augmented in LPS-treated *Spred-2^−/−^* mice. The enhanced acute lung inflammation was reduced when *Spred-2^−/−^* mice were treated with U0126, an ERK-MAPK inhibitor, which was associated with decreased cytokine and chemokine responses. These findings suggest that Spred-2 negatively regulates LPS-induced acute lung inflammation by inhibiting the ERK-MAPK pathway.

The lung is exposed to various pathogens and environmental stimuli that can cause infections and inflammation. Alveolar macrophages are an important component of host defense against invading microorganisms. They play a critical role in initiating and resolving inflammation by releasing many different inflammatory mediators, including cytokines and chemokines [26], [27]. To understand the underlying mechanism for the increased inflammatory response in *Spred-2^−/−^* mice, we hypothesized that alveolar macrophages could be responsible for the enhanced acute lung inflammation. Indeed, alveolar macrophages form *Spred-2^−/−^* mice produced significantly higher levels of TNF-α, CXCL2 and CCL2. *Spred-2* knock down in RAW264.7 cells resulted in decreased levels of TNF-α, CXCL2 and CCL2. Thus, it appears that macrophages contribute to the enhanced LPS-induced cytokine and chemokine response in the absence of Spred-2, likely accounting for the increased LPS-induced acute lung inflammation in *Spred-2^−/−^* mice.

Another possible cell type responsible for the enhanced inflammatory response in *Spred-2^−/−^* mice is the lung epithelial cell, a cell type that is persistently exposed to microorganisms and environmental stimuli [28]. We found that ERK was phosphorylated not only in alveolar macrophages, but also in bronchial epithelial cells. We showed that *Spred-2* knock down MLE-12 cells produced higher levels of CCL2, but not TNF-α or CXCL2, when compared to control cells. These results suggest that the cytokine and chemokine response provoked by LPS varies between different cell types and that epithelial cells also contribute to the enhanced acute lung inflammation by augmenting the chemokine response. It has been demonstrated that alveolar macrophage-derived TNF-α induces alveolar epithelial cells to produce chemokines, resulting in subsequent lung injury [23], [29]. Thus, alveolar macrophages appear to interact with epithelial cells and contribute to the evolution of acute lung inflammation.

The molecular pathways that regulate the lung inflammatory response to LPS are complex and include a variety of promiscuously expressed transcription factors [30]. We found that the ERK-MAPK pathway is involved in the progression of LPS-induced acute lung inflammation, and can be negatively regulated by Spred-2. MAPKs constitute a large modular network that regulates many distinct physiological processes. A recent study indicated that the ERK-MAPK pathway could be inhibited by either p38-MAPK or JNK-MAPK [31]. Additionally, multiple levels of crosstalk exist between the PI3K (phosphoinositide 3-kinase)-Akt and Ras-MAPK signaling pathways [32], [33]. A dynamic model of feedback and crosstalk between the MAPK and AKT signaling pathways has been proposed [34]. The expression of several cytokine genes, including TNF-α, is associated with NF-κB activation [35], and it has been shown that the Ras-MEK-ERK pathways are involved in regulating NF-κB/IκB-dependent production of inflammatory mediators [36]. Although Spred-2 can negatively regulate the Ras-Raf-ERK pathway by binding to Ras, thereby inhibiting the phosphorylation of Raf [13], previous studies and our findings suggest that Spred-2 alters LPS-induced acute lung inflammation indirectly by affecting other signaling pathways. Further studies will be necessary to identify the precise molecular mechanisms involved.

There are other several concerns that were not addressed in this study. First, because U0126 inhibits MEK1 and MEK2 with negligible effects on other protein kinases, such as ERK, p38, and JNK [37], an inhibitor other than U0126 and/or a direct inhibitor against each MAPK needs to be used to elucidate the precise mechanisms involved. Second, the cells expressing Spred-2 in the lung have not yet been characterized because there are no effective antibodies yet available for use in immunohistochemistry. Third, although our data suggested the involvement of alveolar macrophages and bronchial epithelial cells in LPS-induced acute lung inflammation, the degree of contribution to the inflammation by these cell types remains unknown. Fourth, the cytokine and chemokine response in *Spred-2^−/−^* mice was decreased in an initial phase while increasing at the peak of leukocyte infiltration. It is possible that the decreased IL-10 level in an initial phase may contribute to the later phase of cytokine response, but there could be other mechanism(s) involved. Finally, as we successfully overexpressed *Spred-2* in RAW264.7 cells and demonstrated that this resulted in reduced cytokine production *in* *vitro*, the possibility is raised that LPS-induced acute lung inflammation could be treated by Spred-2 supplementation *in* *vivo*. Further studies will be necessary to address these points.

In conclusion, we have shown that Spred-2 deficiency exacerbates the inflammatory response in a murine model of LPS-induced acute lung inflammation. Spred-2 controls the development of LPS-induced lung inflammation by negatively regulating the ERK-MAPK pathway. Although supportive therapy has marginally improved survival of ALI/ARDS patients, effective therapeutic agents that improve clinical outcomes are urgently needed [1], [2]. LPS-induced lung injury is widely used as an experimental model to investigate the mechanisms of ALI [38]. Gaining a better understanding of the signaling pathways involved in this animal model may lead to novel insights and the identification of potential therapeutic targets. Our work presented here suggests that Spred-2 represents such a potential therapeutic target for the treatment of clinical ALI/ARDS.

## Supporting Information

Figure S1 **The production of cytokine and chemokines by bone marrow-derived macrophages.** Bone marrow cells were isolated from femurs and tibias of WT and *Spred-2^−/−^* mice (n = 4) and then were differentiated into bone marrow-derived macrophages after approximately 10 days of culture in L929-conditioned media. Cells from untreated WT (open column) and *Spred-2^−/−^* mice (closed column) were stimulated with LPS (100 ng/mL) for 24 h. TNF-α, CXCL2 and CCL2 were measured by ELISA. #*P*<0.01, vs. WT control.
(TIF)
