# Supplementary figures and images for: Spred-2 Deficiency Exacerbates Lipopolysaccharide-Induced Acute Lung Inflammation in Mice
Source: PLoS One. 2014 Oct 2;9(10):e108914. doi: 10.1371/journal.pone.0108914 (PMC4183529; doi:10.1371/journal.pone.0108914)

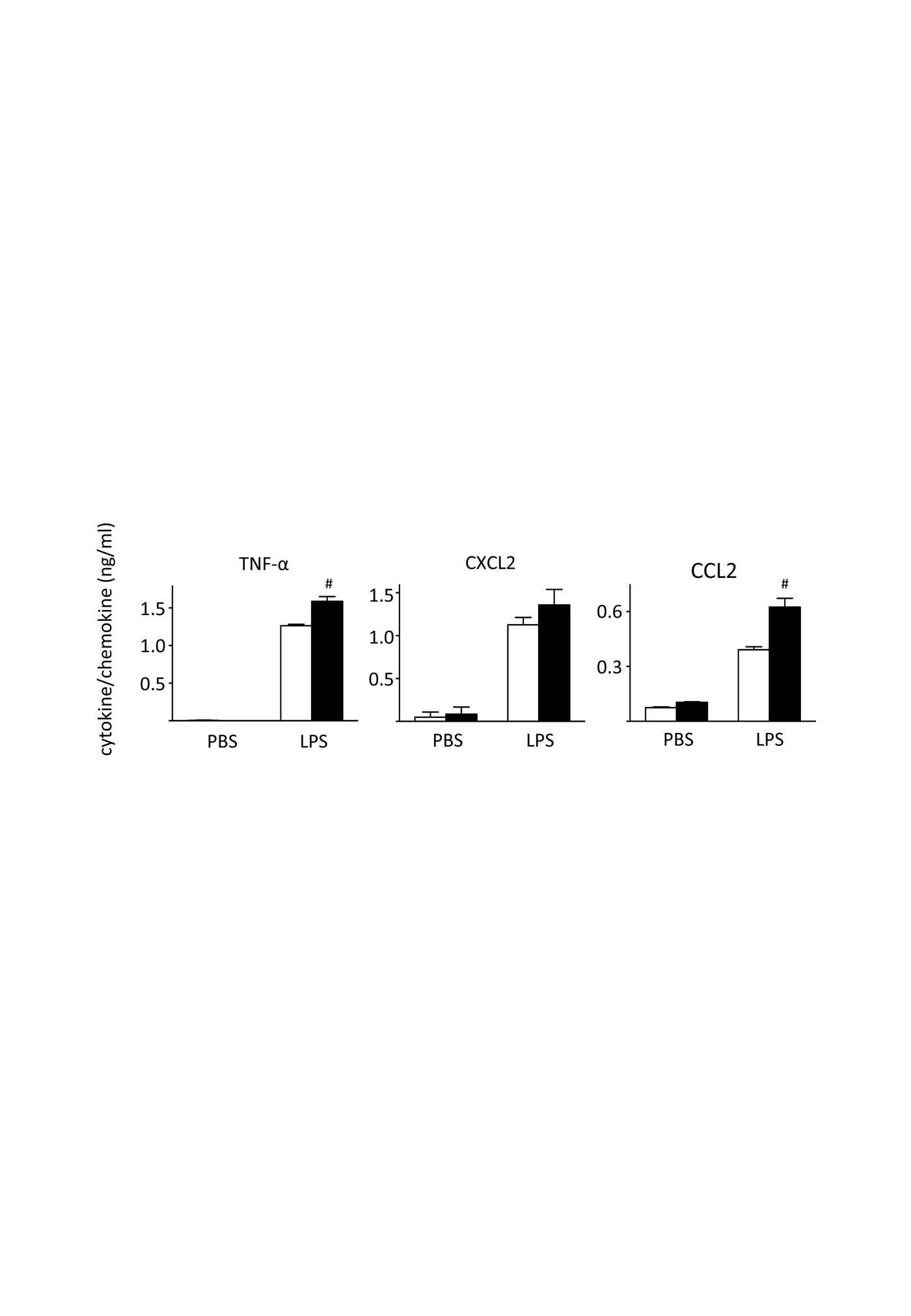

Supplement: Figure S1 — The production of cytokine and chemokines by bone marrow-derived macrophages. Bone marrow cells were isolated from femurs and tibias of WT and Spred-2−/− mice (n = 4) and then were differentiated into bone marrow-derived macrophages after approximately 10 days of culture in L929-conditioned media. Cells from untreated WT (open column) and Spred-2−/− mice (closed column) were stimulated with LPS (100 ng/mL) for 24 h. TNF-α, CXCL2 and CCL2 were measured by ELISA. #P<0.01, vs. WT control. (TIF) [file pone.0108914.s001.tif]
